# Expression of the Chitinase Family Glycoprotein YKL-40 in Undifferentiated, Differentiated and Trans-Differentiated Mesenchymal Stem Cells

**DOI:** 10.1371/journal.pone.0062491

**Published:** 2013-05-09

**Authors:** Daniel J. Hoover, Viola Zhu, Ru Chen, Ken Briley, Pranela Rameshwar, Stanley Cohen, Frederick D. Coffman

**Affiliations:** 1 Center for Biophysical Pathology, UMDNJ-NJMS, Newark, New Jersey, United States of America; 2 Department of Medicine, Division of Hematology/Oncology, BU Medical Center, Boston, Massachusetts, United States of America; 3 Ethicon Endo-Surgery, Cincinnati, Ohio, United States of America; 4 Department of Microbiology, University of Pennsylvania, Philadelphia, Pennsylvania, United States of America; 5 Department of Medicine, UMDNJ-NJMS, Newark, New Jersey, United States of America; 6 Department of Pathology and Laboratory Medicine, UMDNJ-NJMS, Newark, New Jersey, United States of America; National Institutes of Health, United States of America

## Abstract

The glycoprotein YKL-40 (CHI3L1) is a secreted chitinase family protein that induces angiogenesis, cell survival, and cell proliferation, and plays roles in tissue remodeling and immune regulation. It is expressed primarily in cells of mesenchymal origin, is overexpressed in numerous aggressive carcinomas and sarcomas, but is rarely expressed in normal ectodermal tissues. Bone marrow-derived mesenchymal stem cells (MSCs) can be induced to differentiate into various mesenchymal tissues and trans-differentiate into some non-mesenchymal cell types. Since YKL-40 has been used as a mesenchymal marker, we followed YKL-40 expression as undifferentiated MSCs were induced to differentiate into bone, cartilage, and neural phenotypes. Undifferentiated MSCs contain significant levels of YKL-40 mRNA but do not synthesize detectable levels of YKL-40 protein. MSCs induced to differentiate into chondrocytes and osteocytes soon began to express and secrete YKL-40 protein, as do *ex vivo* cultured chondrocytes and primary osteocytes. In contrast, MSCs induced to trans-differentiate into neurons did not synthesize YKL-40 protein, consistent with the general absence of YKL-40 protein in normal CNS parenchyma. However, these trans-differentiated neurons retained significant levels of YKL-40 mRNA, suggesting the mechanisms which prevented YKL-40 translation in undifferentiated MSCs remained in place, and that these trans-differentiated neurons differ in at least this way from neurons derived from neuronal stem cells. Utilization of a differentiation protocol containing β-mercaptoethanol resulted in cells that expressed significant amounts of intracellular YKL-40 protein that was not secreted, which is not seen in normal cells. Thus the synthesis of YKL-40 protein is a marker for MSC differentiation into mature mesenchymal phenotypes, and the presence of untranslated YKL-40 mRNA in non-mesenchymal cells derived from MSCs reflects differences between differentiated and trans-differentiated phenotypes.

## Introduction

The human chitinase family glycoprotein YKL-40 (CHI3L1) has been implicated in tissue remodeling, angiogenesis, and cell survival in both normal and pathological conditions [1]–[4]. YKL-40 protein is synthesized primarily by classically activated macrophages [5], neutrophils [6], *ex vivo* cultured chondrocytes [7], end stage osteoblasts and primary osteocytes [8], osteoblasts treated with TNF-α or IL-1 [9], and in lower amounts by other tissues of mesenchymal origin.

YKL-40 protein is present in normal human serum at concentrations in the low nanomolar range [10]. It is elevated in the serum of patients and in the affected cells of a number of non-cancerous pathological conditions including rheumatoid arthritis [11], asthma [12], and hepatic fibrosis/cirrhosis [13]–[15]. YKL-40 is also synthesized and secreted by a number of human tumors, including tumors of the breast, bone, colon, thyroid, liver, prostate, ovaries, and lung [3], [4], [14]. Further, YKL-40 serum protein levels were directly correlated with morbidity and or mortality in patients suffering from cancers of the breast [16]–[18], colon [19], [20], ovaries [21], and brain [22]. YKL-40 is expressed in a limited number of brain cancers, and is the most highly upregulated gene in high grade glioblastoma multiformae. YKL-40 expression in these high grade glioblastoma multiformae appears linked to the degree of tumor vascularization [23].

Mesenchymal stem cells (MSCs) are multipotent adult stem cells derived primarily from bone marrow, first described as being capable of differentiating into adipocytes, chondrocytes, myoblasts and osteocytes *in vitro* under defined culture conditions [24]. MSCs have more recently been shown to be able to transdifferentiate into cells of endodermal and ectodermal lineage [25]–[28], as well as functional neurons [29], thus MSCs are a relevant system in which to study the molecular details of lineage-specific differentiation. When injected into animal models, MSCs have facilitated the repair and regeneration of damaged tissue types, which has spurred the use of MSCs in clinical trials for a number of disease states, including myocardial ischemia, myocardial infarction, spinal cord injury, stroke, multiple sclerosis, Type I and II diabetes, cirrhosis, kidney transplants, chronic obstructive pulmonary disease, cartilage defects, bone fractures, and graft versus host disease [30].

MSCs differentiate into a number of somatic tissues that express YKL-40, and changes in YKL-40 expression have been noted in differentiation accompanying fetal development in osteogenic and chondrogenic cell lineages [31]. YKL-40 has also been used as a mesenchymal marker in fully differentiated tissues, so we were interested in how YKL-40 is expressed during the formation of these differentiated phenotypes. To this end we examined YKL-40 expression as undifferentiated MSCs were differentiated into osteogenic and chondrogenic phenotypes, as well as during trans-differentiation into neurons. Our specific interest was how YKL-40 expression patterns changed with the differentiation processes, to gain insight into the potential roles of YKL-40 in the mature phenotypes and perhaps in the differentiation processes.

## Materials and Methods

### Reagents and Antibodies

All chemicals were purchased from Sigma-Aldrich (St. Louis, MO) unless otherwise noted. Polyclonal Rabbit anti-YKL-40 antibody was purchased from Quidel (San Diego, CA). Polyclonal chicken anti-Neuron Specific Enolase, Polyclonal chicken anti-β-Tubulin III, Polyclonal rabbit anti-NeuN, Polyclonal rabbit anti-Collagen II, and rabbit anti-chicken HRP antibodies were purchased from Millipore (Temecula, CA). Monoclonal mouse anti-CD44 antibodies were purchased from R&D Systems (Minneapolis, MN). Monoclonal mouse anti-OC4-30 (Osteocalcin) antibodies were purchased from Abcam (Cambridge, MA). Goat anti-rabbit HRP conjugate antibody was purchased from Bio-Rad Laboratories (Hercules, CA). Horse anti-mouse HRP antibodies were purchased from Cell Signaling Technology (Danvers, MA). Mouse monoclonal anti-β-actin HRP conjugate antibody was purchased from Sigma-Aldrich.

### Cell Culture

Mesenchymal stem cells were purchased from Lonza (Allendale, NJ). All other cell lines were purchased from the American Type and Culture Collection (ATCC, Manassas, VA) and cultured in typical culture conditions “TCC” of DMEM media Mediatech Inc. (Manassas, VA) containing 10% FBS, Hyclone (South Logan, Utah), and 0.292 mg/ml L-Glutamine, Mediatech (Manassas, VA) unless otherwise stated. All cell cultures were grown in vented tissue culture flasks from Corning®, (Corning, NY), or tissue culture chamber slides from Lab-Tek/Nalge Nunc (Naperville, IL) for staining.

### Neuronal Differentiation Protocol 1

MSCs of passage 2–5 were differentiated into neurons by first growing them in TCC to 20% confluence, approximately 100,000 cells per tissue culture dish. Then MSCs were exposed for 7, 14 and 30 days to neuronal induction media: Ham’s DMEM/F12, (Mediatech Inc.), 2% FBS, 2% B27 supplement, Invitrogen (Carlsbad, CA), 12.5 ng/ml βFGF (Invitrogen), and 20 µM ATRA, with media changes every 3 to 4 days. This protocol was established by Greco and Rameshwar who demonstrated that this procedure resulted in cells that expressed neural markers, synthesized, packaged and secreted neurotransmitters, and produced post-synaptic currents [29].

### Neuronal Differentiation Protocol 2

MSCs of passage 2–5 were differentiated into neurons by first growing them in TCC to 20% confluence, approximately 100,000 cells per tissue culture dish. Cells were then pre-induced with DMEM, 20% FBS and 1 mM β-mercaptoethanol (β-ME) for 24 hours. The pre-inducement media was then removed, the cells washed with PBS, and differentiation media composed of: DMEM and 10 mM β-ME was added. The cells were then incubated for 24 hours [32].

### Chondrocyte Differentiation using Micromass Pellet Culture (Micropellet)

MSCs of passage 2–5 were removed from culture using a 5 mM EDTA PBS wash, followed by trypsinization. Cells were resuspended in chondrocyte differentiation media at a density of 100,000 cells/10 µl. 10 µl drops were seeded (one 10 µl drop per chamber) into a tissue culture chamber slide and placed in the cell incubator at 37°C, 5% CO2 for 2.5 hours to form 3D cell aggregates. Then chondrocyte differentiation media was gently added to each chamber. Chondrocyte differentiation media consisted of: α-MEM, Lonza (Verviers, Belgium) with 0% FBS, 100 nM dexamethasone, 10 ng/ml TGF β-3, R&D Systems (Minneapolis, MN) & Cell Signaling (Danvers, MA), SITE liquid media supplement, Sigma-Aldrich (as per manufacturer’s instructions 1∶100), 100 µg/ml Sodium Pyruvate (Sigma-Aldrich), and 1.5 mg/ml BSA (Sigma-Aldrich). Media was replenished every 3 days, and cells were harvested after 28 days [33].

### Osteoblast Differentiation

MSC’s of passage 2–5 were grown in TCC until 90% confluent (500,000–600,000 per T75 flask). Then α-MEM (Lonza) with 10% FBS, 50 µg/ml ascorbic acid was added on day ‘0′. After 7 days, 3 mM β-glycerol phosphate was added to the existing protocol and the cells exposed for 23 days, with media changes every 3 to 4 days [34].

### Isolation of RNA

The initial RNA isolation utilized the RNeasy Minikit from Qiagen, following the manufacturer’s instructions. For all subsequent RNA isolations, Trizol was added and cells were lysed directly in the flask (after media removal). The resulting lysate was transferred to 1.5 ml eppi tubes. Samples were then either used or stored at –80°C. 1 ml of each sample of Trizol lysate was then vortexed, and centrifuged at 12,000 g for 10 minutes at 4°C. Cleared supernatants were then moved to new 1.5 ml eppi tubes. Samples were then incubated for 5 minutes at room temperature to allow nucleoprotein dissociation. For phase separation, 0.2 ml of chloroform was added and the tube shook vigorously for 15 seconds, then incubated at room temperature for 3 minutes. Samples were centrifuged at 12,000 g for 15 minutes at 4°C. The clear aqueous phase was moved to a new 1.5 ml eppi tube and RNA precipitation was performed. For RNA precipitation, 5 mg of RNase free glycogen was added to act as an RNA carrier. Then 0.5 ml of 100% isopropanol was added and the sample was incubated at room temperature for 10 minutes. The samples were then centrifuged at 12,000 g for 10 minutes at 4°C. The supernatant was discarded and the resulting RNA pellet was washed two times with 75% ethanol diluted with DEPC treated water. The pellet was then dried for 5 minutes under a laminar flow hood and resolublized in 20 ml of nuclease free water and assessed by the A260/280 ratio method.

### Reverse Transcription-PCR

cDNA was produced following the instructions for the Omniscript Reverse Transcription Kit (Quiagen, Valencia, Ca) utilizing 1 µg of template RNA, 10 mg of Oligo (dT)16 primers (Applied Biosystems, Foster City, CA) and 20 units of RNase Inhibitor (Applied Biosystems) per reaction. (In the initial screening assay, Random Hexamer Primers (Quiagen) were utilized in reverse transcription; all subsequent procedures utilized Oligo (dT)16 primers.) PCR products were then produced using the HotStar Taq Plus PCR kit (Quiagen).

### Polymerase Chain Reaction

The HotStar HiFidelity Polymerase Chain Reaction kit (Qiagen) was used to amplify cDNAs produced during the RT reaction. Kit directions were followed using 2–5 µl RT reaction products and 0.5 µM of each primer in a total volume was 50 µl per sample (primer sequences shown in Table 1; all primers were made by the Molecular Resource Facility (MRF) of the New Jersey Medical School). For all primer pairs except GAPDH, the amplification cycles consisted of an activation step of 5 minutes at 95°C, then 35 cycles of: (30 seconds at 94°C, 30 seconds at 55°C, 1 minute at 72°C), followed by a clean up step of 10 minutes at 72°C and a 4°C indefinite hold. For GAPDH, the times and temperatures were the same except the temperature of the annealing step was 62°C. Samples removed from the thermocycler were either refrigerated and analyzed by agarose gel electrophoresis within a few days or stored at −20°C.

**Table 1:** PCR primer sequences.

Primers:		NCBI Ref. Seq.	Amplicon Length (bp)
β-actin	Forward: 5′ – ATG TTT GAG ACC TTC AAC AC –3′	NM_001101.3	495
	Reverse: 5′ – CAC GTC ACA CTT CAT GAT GG –3′		
Chitinase	Forward: 5′ – CTC TCT GGG CAG GTG TAG TGG –3′	NM_004000.2	328
	Reverse: 5′ – GTA GAA GAA TCC ACC ATA GGG T –3′		
Chitotriosidase	Forward: 5′ – GAG GCT GGG CCC AGG ATC AC –3′	NM_001270509.1	193
	Reverse: 5′ – GAA GAG GGG CAC AAA CCA AAG –3′		
GAPDH	Forward: 5′ – CCA CCC ATG GCA AAT TCC ATG GCA –3′	NM_002046.3	598
	Reverse: 5′ – TCT AGA CGG CAG GTC AGG TCC ACC –3′		
IKB	Forward: 5′ – CTG AAG AAG GAG CGG CTA CTG G –3′	NM_020529.2	322
	Reverse: 5′ – CAA TTT CTG GCT GGT TGG TGA T–3′		
MIF 1	Forward: 5′ – CTC TCC GAG CTC ACC CAG CAG –3′	NM_002415.1	255
	Reverse: 5′ – CGC GTT CAT GTC GTA ATA GTT –3′		
MIF 2	Forward: 5′ – AGC CTG CAC AGC ATC GGC AAG ATC G –3′	NM_002415.1	269
	Reverse: 5′ – TAT TTC TCC CCA CCA GAA GGT TGG –3′		
YKL-40	Forward: 5′ – TGA GGC ATC GCA ATG TAA G –3′	NM_001276.2	266
	Reverse: 5′ – AAG GGG AAG TAG GAT AGG GG –3′		

### Agarose Gel Electrophoresis

Samples (25 µl each) were run on a 2% agarose gel in TAE at 50v, using a 5x DNA loading buffer consisting of glycerol (3% v/v) and Fast Orange dye (1% w/v). The gel was then trimmed and stained with ethidium bromide in 1x TAE for ½ an hour, destained three times for 5 minutes each, using approximately 200 ml of ddH2O and visualized in UV using a Syngene transilluminator and Genesnap software from Synoptics (Frederick, MD).

### Western Blot Analysis

Cells were washed three times in ice-cold phosphate buffered saline (PBS) and were lysed with cell lysis buffer (Cell Signaling, Danvers, MA), containing Roche Complete protease inhibitor (Roche Diagnostics, Mannheim, Germany). The cell lysate was then spun at 10,000 g for 20 minutes to remove insoluble cellular materials. The supernatant was then removed to fresh 1.5 ml eppendorf tubes and the protein concentration assessed using the Bio-Rad Protein Assay. SDS-PAGE sample buffer with β-mercaptoethanol was then added to the protein extract and the combination was incubated for 5 minutes at 100°C. Samples were then used immediately or stored at –80°C. SDS-PAGE electrophoresis was then performed using precast 4–20% gradient Tris-HCl gels (Bio-Rad). All gels were pre-run in the presence of a running buffer containing SDS for one hour just prior to use. After gel electrophoretic protein separation, the gel was then blotted overnight onto a PVDF membrane (Bio-Rad), then incubated in a solution of 5% milk in TBST (Tris buffered saline with Tween: 50 mM Tris-HCl, 137 mM NaCl, 0.1% Tween 20) to prevent non-specific binding. The blot was then incubated with a rabbit anti-YKL-40 (1∶1,000) antibody (Quidel), followed by Goat anti-rabbit HRP conjugate (1∶5,000) secondary antibody (Sigma). A Coomassie blue gel stain was performed to confirm equal total protein loading. Occasionally mouse monoclonal anti-β-actin HRP conjugate antibody (1∶10,000) blotting was performed on a membrane subsequent to stripping with Restore Western Blot Stripping Buffer, Pierce/Thermo Scientific (Rockford, IL) to further confirm protein loading.

### Alcian Blue Stain

Chondrocyte like cells produced in the microdot method were first treated with 4% paraformaldehyde/PBS (without magnesium or calcium). 100 µl of 4% paraformaldehyde/PBS was added to the existing 200 µl of chondrocyte differentiation media and incubated at room temperature for 5 minutes. Then all fluid was gently aspirated away, and 200 µl of 4% paraformaldehyde/PBS was added to the microdot and incubated at room temperature for 1 hour. The microdot was then washed 2x in PBS and incubated with Alcian Blue 8Gx stain solution overnight at room temperature. Alcian Blue 8Gx stock stain solution consists of: 8 mg of Alcian Blue 8Gx added to a solution of 30 ml of 100% ethyl alcohol and 20 ml glacial acetic acid. The stain solution was then aspirated and the microdot was incubated for 20 minutes with destain solution 2x. Destain stock solution consists of: 30 ml of 100% ethyl alcohol and 20 ml glacial acetic acid. 200 µl of PBS was added after destain solution was aspirated away, and the microdot was imaged. Alcian blue 8Gx stains glycosaminoglycans and mucopolysaccharides to a pale blue color.

### Alizarin Red S Stain

Osteoblast like cells were first treated with 4% paraformaldehyde/PBS (without magnesium or calcium). 100 µl of 4% paraformaldehyde/PBS was added to the existing 200 µl of osteoblast differentiation media and incubated at room temperature for 5 minutes. Then all fluid was gently aspirated away, and 200 µl of 4% paraformaldehyde/PBS was added to the cell culture and incubated at room temperature for 1 hour. The culture was then washed 2x in PBS and incubated with Alizarin Red S stain solution for 45 minutes in the dark at room temperature. The stain solution was then aspirated and the culture was washed 2x in PBS taking care to not remove any crystals. The culture was then imaged. Alizarin Red S stain solution consists of: 0.2 g of Alizarin Red S dissolved in 10 ml of ddH2O. Just prior to use, the pH of the Alizarin Red S solution was adjusted to pH 5 using NaOH.

## Results

### Undifferentiated MSCs make YKL-40 mRNA but not YKL-40 Protein

**Figure 1:**
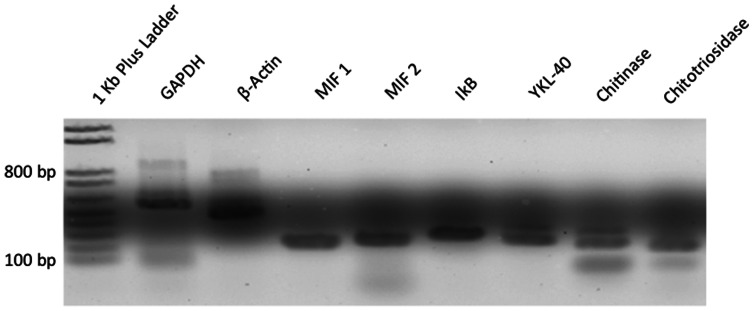
Screening RT-PCR analysis of transcribed mRNAs in undifferentiated MSCs. Total RNA was isolated from undifferentiated mesenchymal stem cells cultured in tissue culture plates using Trizol. Following reverse transcription using random hexamer primers, the resulting cDNAs were amplified in a series of PCR reactions using primers for YKL-40 and other members of the human chitinase family, as well as primers for two control mRNAs (beta actin and GAPDH), the cytokine MIF, and the NF-kB inhibitor IkB (primer sequences shown in Table 1). PCR reaction products were separated using agarose gel electrophoresis and visualized using EtBr and UV light.

**Figure 2:**
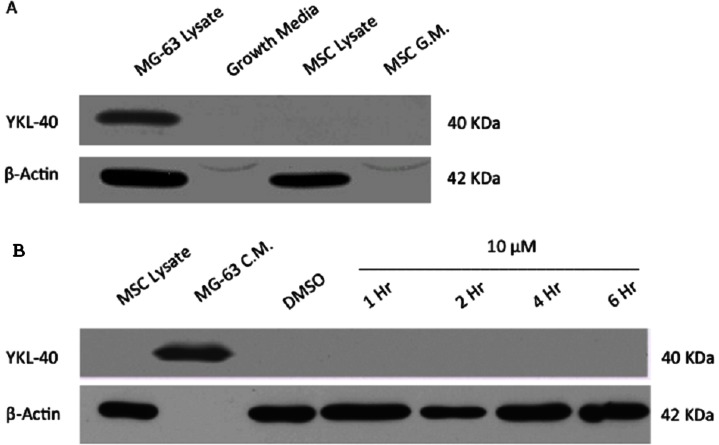
Western blot analysis of YKL-40 in the lysate and conditioned media from undifferentiated MSCs. **A.** Western blot analysis using a primary anti-YKL-40 polyclonal antibody of a gel containing a positive YKL-40 control (conditioned media from MG-63 osteosarcoma cells), MSC media alone, cell lysate from undifferentiated MSCs, and conditioned media from undifferentiated MSCs. No YKL-40 protein was detected in either the MSC cell lysate or MSC conditioned media. **B.** Western blot of cell lysates from undifferentiated MSCs treated with 10 µM of the proteasome inhibitor MG132 for 1, 2, 4, and 6 hours. Controls include DMSO alone (equivalent to that in the MG132 stock solution) and MG-63 conditioned media as a positive control for YKL-40.

Undifferentiated MSCs have been reported to express YKL-40 mRNA, so initial experiments were performed to establish the levels of YKL-40 mRNA and the corresponding levels of YKL-40 protein synthesized by undifferentiated mesenchymal stem cells [35], [36]. As shown in Figure 1, MSCs transcribe significant quantities of YKL-40 mRNA, along with mRNA for the related chitinase family proteins chitinase and chitotriosidase, as well as for MIF (Macrophage migration inhibitory factor). Surprisingly, western blots revealed the absence of YKL-40 protein in both the undifferentiated MSC cell lysate and cell culture media (Figure 2A). To insure that YKL-40 was not being translated and then rapidly degraded by the proteasome, undifferentiated MSCs were incubated with the proteosome inhibitor MG132 for up to 6 hours, however western blot analysis demonstrated that YKL-40 protein was not present in either the cell lysate or media supernatant at any time within this 6 hour period (Figure 2B). Thus undifferentiated MSCs transcribe measurable amounts of YKL-40 mRNA, but that message is not translated into protein.

### MSCs Differentiated into Osteoblasts and Chondrocytes Synthesize YKL-40 Protein. MSCs Trans-differentiated into Neurons using bFGF and ATRA do not Synthesize YKL-40 Protein but Still Transcribe YKL-40 mRNA

**Figure 3:**
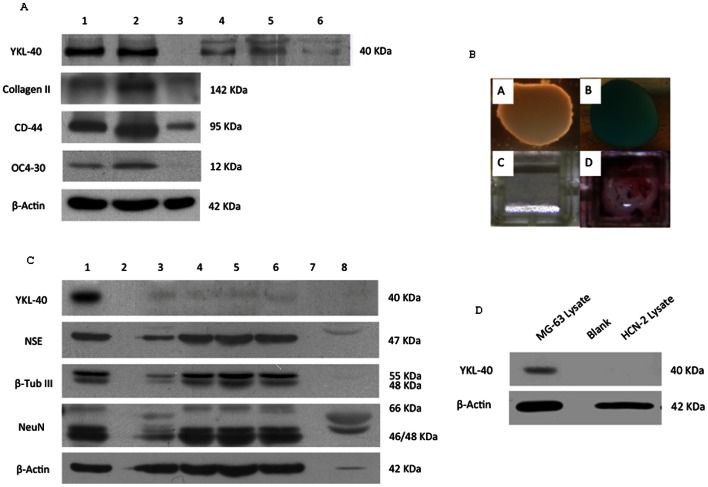
YKL-40 protein expression in differentiated/trans-differentiated MSC phenotypes. **A.** Western blots of cell lysates (lanes 1–3) and culture media (lanes 4–6) from MSCs differentiated into osteoblasts (lanes 1 and 4), chondrocytes (lanes 2 and 5), and undifferentiated MSCs (lanes 3 and 6) for the presence of YKL-40 and the differentiation markers Type II collagen, CD-44, and osteocalcin. **B.** Top Panel (A, B): Alcian Blue Stain of differentiated chondrocytes produced in the microdot method (A): negative control, (B): Differentiated chondrocytes. The pale blue color indicates the presence of glycosaminoglycans and mucopolysaccharides, and is the classic result for this cytology stain identifying chondrocytes. Bottom Panel (C, D): Alizarin Red S Stain for calcium. (C): Negative control, (D): Osteoblasts differentiated from MSC’s. Red crystals represent the deposition of calcium indicative of osteoblast activity. **C.** Western blot of YKL-40 protein in MSC lysate of neural trans-differentiation protocol 1. Lane 1, MG-63 lysate (positive control). Lane 2, empty. Lane 3, Untreated MSC lysate, (negative control). Lanes 4–6, lysate from MSCs treated with protocol 1 for 7 days, 14 days, and 30 days, respectively. Lane 7, empty. Lane 8, media control. Neuron markers: Neuron Specific Enolase (NSE), β-Tubulin III, and NeuN are shown. Splice variants or protein isoforms have their individual molecular weights listed (with one exception: the light 66 kDa band that appeared following incubation with anti-NeuN antibody is identified by the antibody manufacturer as an uncharacterized cross-reactive species). **D.** Western blot of YKL-40 protein in HCN2 cell lysates (non-cancerous neuronal cell line) grown in normal culture conditions.

**Figure 4:**
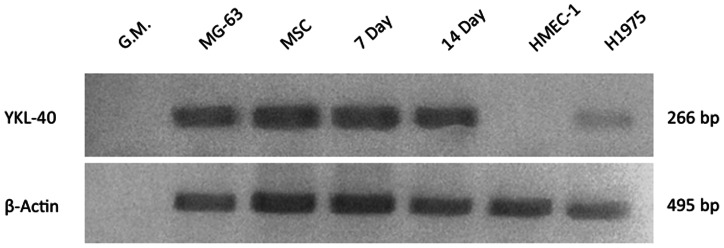
RT-PCR analysis of transcribed mRNAs in mesenchymal stem cells trans-differentiated into neurons using bFGF and ATRA. MSCs were differentiated using Neuron Transdifferentiation Protocol 1, and RT-PCR was performed using primers for YKL-40 (top) and beta actin (bottom). Lane 1, growth media. Lane 2, RNA from MG-63 cells (positive control). Lane 3, RNA from undifferentiated MSCs. Lane 4: RNA from neuron like cells trans-differentiated for 7 days. Lane 5∶14 days. Lane 6, RNA from HMEC1 microvascular endothelial cells. Lane 7, RNA from H1975 non-small cell lung cancer cells.

Upon differentiation into osteoblast and chondrocyte lineages, significant amounts of YKL-40 protein appeared in both the cell lysates and media supernatants, demonstrating that YKL-40 protein was both synthesized and secreted by these two differentiated phenotypes (Figure 3A). Protein marker expression was consistent with previous reports: type II collagen is a strong chondrocyte marker but is also expressed in osteocyte progenitors, CD44 is a cartilage marker but is also expressed by osteocytes and osteoclasts, and the bone marker osteocalcin is also expressed in arthritic chondrocytes. However, the differentiated chondrocytes and osteocytes both demonstrated classic phenotype-specific staining with Alcian Blue and Alizarin Red, respectively (Figure 3B). However, MSCs trans-differentiated into neurons using ATRA and bFGF (protocol I) did not synthesize or secrete YKL-40 protein even after 30 days (Figure 3C). A number of neuronal marker proteins were upregulated during this procedure, in agreement with previous studies that established significant parallels between these trans-differentiated neurons and normal neurons, including neuronal marker expression, neurotransmitter synthesis and release, and the presence of post-synaptic currents [29]. YKL-40 protein was similarly absent in the neuronal cell line HCN2 (Figure 3D). Thus neither the trans-differentiated neuronal cells nor the cell line derived from normal neurons express YKL-40 protein, in agreement with the general absence of YKL-40 protein in most CNS tissues. However, RT-PCR demonstrated that the level of YKL-40 mRNA in the bFGF/ATRA trans-differentiated MSCs remained essentially identical to that seen in undifferentiated MSCs, even two weeks after the initiation of differentiation (Figure 4). This suggests that the mechanism which suppresses the translation of YKL-40 mRNA in the undifferentiated MSCs remains in force when MSCs are trans-differentiated into neurons using this protocol.

### MSCs Trans-differentiated using ßME Express but do not Secrete YKL-40 Protein

**Figure 5:**
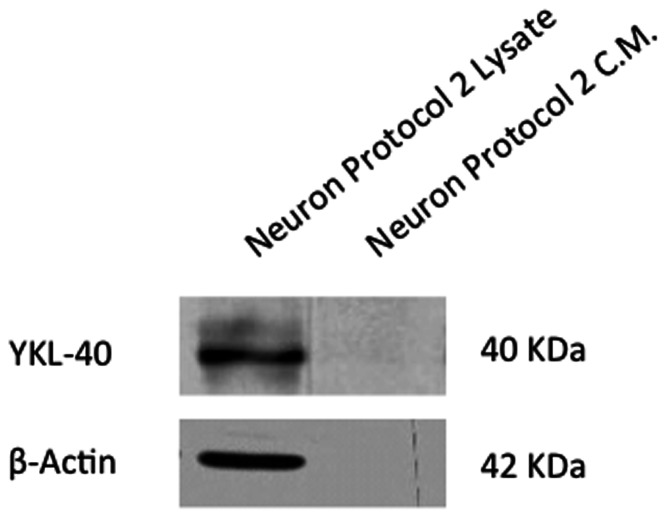
Western Blot analysis of YKL-40 in cell lysate and media supernatant of MSCs differentiated using neuron protocol 2 (using ß-mercaptoethanol). Lane 1, cell lysate. Lane 2, conditioned media from trans-differentiated cell cultures.

ß-mercaptoethanol has been used as a differentiating agent and can stimulate MSC differentiation into a phenotype that has some neural characteristics [32]. MSCs differentiated using BME (protocol II) express YKL-40 protein in the cell lysate at a level comparable to that seen in the cell lysates of the differentiated osteoblasts and chondrocytes, but no YKL-40 protein was observed in the media supernatant (Figure 5). Thus MSCs differentiated using this protocol synthesize YKL-40 protein but do not secrete it, an unusual behavior not seen in normally differentiated cells.

## Discussion

YKL-40 has been utilized as a mesenchymal marker in numerous gene expression array studies, including those of stem cell differentiation and epithelial to mesenchymal transition events in normal and tumor cells [2], [23], [37]. The presence of YKL-40 mRNA in undifferentiated MSCs has been previously documented [35], [36], however the absence of YKL-40 protein translation by undifferentiated MSCs was unexpected and represents an interesting new finding.

One obvious reason why undifferentiated MSCs would store but not translate an mRNA is the protein coded by that mRNA is required for processes in the very early stages of differentiation, and thus having readily available mRNA would allow cells to quickly express that particular protein. Since cells can easily synthesize new proteins within an hour of an initial stimulus, untranslated mRNAs that are stored in cells usually encode genes whose products are needed very early in a stimulus-driven process such as in activation, differentiation or cell stress, thus the metabolic cost of storing the dormant mRNA can be justified. However, the YKL-40 mRNA did not appear to be immediately translated in the MSCs stimulated to differentiate into osteocytes or chondrocytes - it took at least 24 hours following the addition of either osteoblast or chondrocyte differentiation media for detectable levels of YKL-40 protein to appear (Hoover, unpublished). While we cannot rule out the requirement for YKL-40 in very early differentiation at levels below our detection threshold, our results suggest that YKL-40 is not needed early in these two differentiation pathways that lead to mature YKL-40-synthesizing phenotypes. YKL-40 protein might be required early in one of the MSC differentiation pathways not examined in this study, such as differentiation into adipocytes, although it would be surprising if YKL-40 played a role in one of those pathways but not in the pathways examined considering the well-documented expression of YKL-40 in bone and cartilage. It is also possible that the YKL-40 mRNA may have important roles completely separate from any roles of the protein, for example, in undifferentiated MSCs the untranslated YKL-40 mRNA may function as a “competing endogenous RNA (ceRNA) and participate in the regulation of the transcriptome as has been recently proposed [38]. These possibilities are intriguing and merit further study.

Our finding that MSCs do not express YKL-40 protein when differentiated into a neuronal phenotype using ATRA and bFGF correlates with the observation that YKL-40 protein is not usually found in normal human CNS neurons, nor generally in normal brain tissues with the exception of low level expression in a subpopulation of microglia and reactive astrocytes [39]–[41]. The ATRA/bFGF/B27 differentiation protocol was previously demonstrated to differentiate MSCs into a phenotype that expressed glial and neuronal progenitor markers (many also assessed in this study in Figure 3C), synthesized, packaged and released neurotransmitters and produced spontaneous post-synaptic currents [29], thus further corroborating the absence of YKL-40 protein production with the neuronal phenotype. While very low levels of YKL-40 mRNA have been reported in homogenates of normal brain tissues [42], the continued presence of high levels of YKL-40 mRNA following ATRA/bFGF/B27 neuronal differentiation suggests that despite the many similarities to mature neurons, some differences exist between normal neurons differentiated from neural stem cells and neurons trans-differentiated from MSCs using this protocol. This also indicates that the mechanisms suppressing YKL-40 translation in undifferentiated MSCs remain active in ATRA/bFGF/B27 differentiated MSCs.

The presence of YKL-40 protein in MSCs following treatment with ß-mercaptoethanol as a trans-differentiating agent (protocol II) suggests that MSCs differentiated using this protocol represent even less of a true neuronal phenotype than those trans-differentiated using the ATRA/bFGF/B27 protocol. While MSCs differentiated using small molecule chemical inducers such as ß-mercaptoethanol and DMSO show increases in the expression of some neuronal markers, they also show increased apoptosis and do not show the electrophysiological properties of neurons in patch clamp experiments [43]. It should be noted that any *in vitro* trans-differentiation protocol may push MSCs to assume a phenotype that is beyond their normal differentiation limits *in vivo*. Trans-differentiation may result in cells with significant epigenetic differences as compared to the normal phenotype, and care must be taken both in interpreting results of studies that push cells well beyond the natural limits of differentiation, and in utilizing trans-differentiated cells for therapeutic applications [44].

The mechanism of suppression of YKL-40 mRNA translation in undifferentiated MSCs remains unknown. A common mechanism of translational suppression is miRNA binding to mRNA 3′ sequences, and reports have documented significant changes in the levels of specific miRNAs in MSC differentiation, including miR-27a, miR-148b and miR-489 in MSC differention into osteoblasts and miR-145 in MSC differentiation into chondrocytes [45], [46]. None of these miRNAs were identified by the major miRNA target search algorithms as being able to target YKL-40 mRNA, and the miRNAs that were listed as being able to target YKL-40 mRNA sequences were not among those identified as being significantly up or downregulated during MSC differentiation. Thus if YKL-40 mRNA is being silenced in undifferentiated MSCs by miRNAs, it is being silenced either by miRNA species that bind in a manner that is not recognized by the current algorithms, or by miRNA species whose total levels do not change appreciably during differentiation but whose available levels change due to significant changes in the levels of other binding partners during osteogenic and chondrogenic differentation.

These results further support the utility of YKL-40 protein expression as a marker for MSC differentiation into mature mesenchymal phenotypes as well as a mesenchymal marker in general. In addition, they suggest that the presence of YKL-40 mRNA is indicative of a mesenchymal or a pre-mesenchymal phenotype, or of a trans-differentiated phenotype with at least one residual mesenchymal characteristic. Although these bFGF/ATRA trans-differentiated neurons appear much like normal neurons – they express multiple neuronal markers, they synthesize, package and secrete neurotransmitters, and they produce post-synaptic currents - the presence of YKL-40 mRNA demonstrates that the trans-differentiated neurons are in at least one way different from normal neurons, which do not contain YKL-40 mRNA. Such differences must be screened and evaluated when trans-differentiated cells are utilized in place of their normally differentiated counterparts.
