# Correction: Expression of the Chitinase Family Glycoprotein YKL-40 in Undifferentiated, Differentiated and Trans-Differentiated Mesenchymal Stem Cells

**DOI:** 10.1371/journal.pone.0096230

**Published:** 2014-04-21

**Authors:** The PLOS ONE Staff

The fourth author’s name is spelled incorrectly. The correct name is: Kenneth Briley Jr.
